# Temporal trends in Human T-Lymphotropic virus 1 (HTLV-1) associated myelopathy/tropical spastic paraparesis (HAM/TSP) incidence in Martinique over 25 years (1986-2010)

**DOI:** 10.1371/journal.pntd.0006304

**Published:** 2018-03-19

**Authors:** Stephane Olindo, Severine Jeannin, Martine Saint-Vil, Aissatou Signate, Mireille Edimonana-Kaptue, Julien Joux, Harold Merle, Pascale Richard, Samuel Granjeaud, Philippe Cabre, Didier Smadja, Raymond Cesaire, Agnes Lezin

**Affiliations:** 1 Department of Neurology, University Hospital of Bordeaux, Bordeaux, France; 2 Department of Neurology, University Hospital of Martinique, Martinique, France; 3 Department of Ophtalmology, University Hospital of Martinique, Martinique, France; 4 Etablissement Français du Sang de Martinique, Martinique, France; 5 Aix-Marseille University, CNRS, INSERM, Institut Paoli Calmettes, CRCM, CIBI Plateform, Marseille France; 6 Department of Neurology, Hospital of Sud-Francilien, Corbeil-Essonnes, France; 7 Department of Virology, University Hospital of Martinique, Martinique, France and EA 4537, Université des Antilles et de la Guyane, Martinique, France; St. Marianna University School of Medicine, Institute of Medical Science, JAPAN

## Abstract

**Background:**

Human T-lymphotropic virus type 1 (HTLV-1) has been discovered in 1980 and has been linked to tropical spastic paraparesis (HAM/TSP) in 1985 in Martinique. There is no data on HAM/TSP incidence trends. We report, in the present work, the temporal trends incidence of HAM/TSP in Martinique over 25 years.

**Methods:**

Martinique is a Caribbean French West Indies island deserved by a unique Neurology Department involved in HAM/TSP diagnosis and management. A registry has been set up since 1986 and patients diagnosed for a HAM/TSP were prospectively registered. Only patients with a definite HAM/TSP onset between 1986 and 2010 were included in the present study. The 25-year study time was stratified in five-year periods. Crude incidence rates with 95% confidence interval (95%CI) were calculated using Poisson distribution for each period. Age-standardized rates were calculated using the direct method and the Martinique population census of 1990 as reference. Standardized incidence rate ratios with 95% CIs and *P* trends were assessed from simple Poisson regression models. Number of HTLV-1 infection among first-time blood donors was retrospectively collected from the central computer data system of the Martinique blood bank. The HTLV-1 seroprevalence into this population has been calculated for four 5-year periods between 1996 and 2015.

**Results:**

Overall, 153 patients were identified (mean age at onset, 53+/-13.1 years; female:male ratio, 4:1). Crude HAM/TSP incidence rates per 100,000 per 5 years (95%CI) in 1986–1990, 1991–1995, 1996–2000, 2001–2005 and 2006–2010 periods were 10.01 (6.78–13.28), 13.02 (9.34–16.70), 11.54 (8.13–14.95), 4.27 (2.24–6.28) and 2.03 (0.62–3.43). Age-standardized 5-year incidence rates significantly decreased by 69% and 87% in 2001–2005 and 2006–2010 study periods. Patients characteristics did not differ regarding 1986–2000 and 2001–2010 onset periods. Between 1996–2000 and 2011–2015 study periods, the HTLV-1 seroprevalence significantly decreased by 63%.

**Conclusion:**

Martinique faces a sudden and rapid decline of HAM/TSP incidence from 2001 in comparison to 1986–2000 periods. Reduction of HTLV-1 seroprevalence, that may result from transmission prevention strategy, could account for HAM/TSP incidence decrease.

## Author summary

Human T-lymphotropic virus type 1 (HTLV-1) was discovered in 1980 and HTLV-1-associated myelopathy/tropical spastic paraparesis (HAM/TSP) was described five years later in 1985. HAM/TSP is a progressive disabling disorder characterized by spastic paraparesis with bladder and bowel dysfunction that constitutes a significant public health problem in endemic areas. Up to date, there is no efficiency treatment of HAM/TSP and prevention of HTLV-1 transmission is critical to limit the disease spreading throughout communities. In the present 25-year-study time, we report a significant decrease of HAM/TSP incidence estimated more than 70% in early 2000 compared to 1986–2000 period in Martinique a French West Indies Island. We found a trend to a significant older age at onset after 2000 (52.1 years versus 57.5 years, p = 0.06) that may reflect an age cohort effect and that could be indicative of a rapid decrease in HTLV-1 seroprevalence. We showed a significant decline in HTLV-1 infection among first-time blood donors between 1996–2000 and 2011–2015 study periods. Thus, probable HTLV-1 seroprevalence decrease secondary to HTLV-1 antibodies screening in blood donors and pregnant women and to iterative information campaigns could partly account for HAM/TSP incidence decline. This study emphasizes the importance of prevention strategies to control HAM/TSP development in HTLV-1 endemic areas.

## Introduction

Human T-lymphotropic virus type 1 (HTLV-1) is associated with many diseases including HTLV-1-associated myelopathy/tropical spastic paraparesis (HAM/TSP). It is estimated that about 10–20 million people are infected with HTLV-1 throughout the world [1].

Whereas HTLV-1 seroprevalence is unknown for 80% of world population [2], data are available in endemic regions and ranges from less than 1 per 10,000 people to more than 10%. The highest rates are found in Japan, Brazil, Colombia, the Caribbean islands, Equatorial Africa, Northeast Australia and Papua New Guinea [3]. Routes of infection include unscreened transfusion [4,5] and organ transplants [6], sharing of needles or syringes with infected subjects, sexual contact [7] and breast-feeding [8,9]. The predominant HTLV-1 horizontal transmission through condom-less sex leads to dramatic seroprevalence regional variations even in high endemic area. Prevalence in population increases steadily with age and is higher in females [10].

The risk of developing HAM/TSP in HTLV-1 infected individuals has been assessed and deeply varies between studies and ethnic groups. In southern Japan the lifetime risk is 0.25% [11] while the 10-year risk reaches 5.3% in a Brazilian cohort [12]. Whereas, HTLV-1 seroprevalence rate is widely reported [3], no data is available on HAM/TSP incidence in general population and in defining area.

We report in the present study the incidence of HAM/TSP and its temporal trends, in the population of Martinique, French West Indies, over a 25-year period from 1986 to 2010. Temporal trends in HTLV-1 infection prevalence among first-time blood donors between 1996 and 2015 have also been calculated.

## Methods

### Study design and population

Martinique is one of the most highly developed islands in the Caribbean, classified high (41st) in terms of global human development at the world level. The population steadily increased between 1982 (328,566) and 2010 (394,171). The great majority (95%) of the population is Afro-Caribbean.

Our Neurology department, located at the University Hospital of Martinique, constitutes the only neurological facility for patients in Martinique. It is highly involved in HAM/TSP diagnosis and management since the original description of the association between HTLV-1 and HAM/TSP [13]. The unique virology department is included in the University hospital. The island is also deserved by 3 neurological rehabilitation centers that work closely with the Neurology department. Since HAM/TSP diagnosis requires cerebrospinal fluid analysis, practitioners and other hospitals use to refer suspected cases to our department. Between 1986 and 2014, the global health care networks implemented for HTLV-1 infection experienced only slight changes. The most significant changes related to care provided in the Neurology and Rehabilitation departments for HAM/TSP symptoms that have improved between 1986 and 2014.

To reduce HTLV-1 transmission among Martinique population, prevention programs were implemented in the early nineties. Blood-donor and organ-donor screening was systematically introduced. Blood and organ donations are invariably rejected if HTLV-1 is detected. Similarly, antenatal screening was implemented to prevent mother-to-child transmissions of HTLV-1 by breastfeeding avoidance. Information campaigns were regularly performed encouraging the use of condom to prevent sexually transmitted infection including HIV and HTLV-1.

The present study is based on a retrospective analysis on data prospectively collected between 1986 and 2015 in a dedicated registry. Patients with a HAM/TSP onset between 1986 and 2010 were considered.

### Case ascertainment and assessment of HAM/TSP patients

A registry has been set up since January 1986 and was still in progress in June 2015. HAM/TSP characteristics were collected carefully and continuously. Physicians involved in HTLV-1 diseases including neurologists, rehabilitation physicians, ophthalmologists, hematologists and dermatologists reported patients included in the registry. On a yearly basis, a cross-analysis of virology and clinical database was performed.

A standardized case report form has been elaborated and collected the demographic data, the medical history, the clinical symptoms, the initial symptoms (gait impairment or urinary disturbances), the onset-to-diagnosis delay and the CSF analysis. The year of first symptoms, such as stiffness or weakness in the legs or urinary disturbances, defined the year of HAM/TSP onset.

Peripheral blood mononuclear cells (PBMCs) were isolated from EDTA-enhanced blood by density gradient centrifugation. Real Time TaqMan PCR was performed on DNA extracted from dry pellets of 10^6^ cells stored at -80°C after preparation from blood sample PBMCs. Forward and reverse primers for HTLV-I DNA quantitation: SK110 (5′-CCCTACAATCCAACCAGCTCAG-3′, HTLV-I nucleotide 4758–4779; SK111 (5′-GTGGTGAAGCTGCCATCGGGTTTT-3′, HTLV-I nucleotide 4943–4920). Internal HTLV-I TaqMan probe: 5′CTTTACTGACAAACCCGACCTACCCATGGA-3′; Located between position 4829 and 4858 of the HTLV-I genome, and carried a 5′ reporter dye FAM (6-carboxy fluorescein) and a 3′ quencher dye TAMRA (6-carboxy tetramethyl rhodamine). Forward and reverse primers for human albumin DNA quantification: Alb-S (5′-GCTGTCATCTCTTGTGGGCTGT-3′) and Alb-AS (5′-AAACTCATGGGAGCTGCT GGTT-3′). Alb TaqMan probe (5′-FAM-CCTGTCATGCCCACACAAATCTC TCC-TAMRA-3′) were used as described previously [14]. Results were expressed as the number of HTLV-1 copies per 10^6^ PBMCs.

For the purpose of the present study, each chart was reviewed and HAM/TSP diagnosis was defined according to an updated staged approach to the World Health Organization criteria [15]. Only definite HAM/TSP was retained in the presence of the following: 1) A non-remitting progressive spastic paraparesis with sufficiently impaired gait to be perceived by the patient. Sensory symptoms or signs can be present. When present, they remain subtle and without a clear-cut sensory level. Urinary and anal sphincter signs or symptoms can be present; 2) Presence of HTLV-1 antibodies in serum and CSF confirmed by western blot analysis and/or a positive PCR for HTLV-1 in blood and/or CSF; 3) Systematic spinal cord imaging and extensive biological work-up ruled out differential diagnosis of progressive paraparesis.

### HTLV-1 seroprevalence assessment among blood donors

In Martinique, all blood donations are performed in the center for blood transfusion (Etablissement Français du Sang). Demographic characteristics of all first-time blood donors tested positive for HTLV-1 were retrospectively collected from the central computer data system of the blood bank. Only donors tested positive with ELISA assay and confirmed with Western blot assay were retained. Data were available for the 20-year period from January 1, 1996 and December 31 2015. Characteristics of all donors were not available for the present study.

### Ethical statement

The present research was conducted according to the principles of the declaration of Helsinki. In 1995, a hospital ethic committee (Comité Consultatif de Protection des Personnes dans la Recherche Biomédicale) was constituted and approved the HAM/TSP registry. Between 1986 and 1994, only oral informed consents were obtained whereas all patients included in the registry after 1994 have given their written informed consent. After standardized information, the oral consent was obtained and collected in the patient’s chart. The hospital ethic committee allowed retrospectively oral consents for 1986–1994 period. For child inclusion participant, a parent has provided informed consent on the child’s behalf. Analysis was performed on anonymized data to ensure confidentiality.

### Statistical analysis

Due to the small number of events per year, the 25-year study time, from January 1, 1986 to December 31, 2010, was stratified in five-year periods for HAM/TSP incidence analysis. Similarly, the 20-year study time, from January 1, 1996 to December 31, 2015, for HTLV-1 seroprevalence in first-time blood donors was stratified in four 5-year periods.

The numerator for calculation of incidence rate was the number of HAM/TSP patients with a disease onset in the defining period. The denominator was based on data provided by French National Institute for Statistical and Economic Studies (www.insee.fr). By convention, the population of the last year of each defined period was considered for calculation. Crude incidence rates with 95% confidence intervals (95%CIs) were calculated using Poisson distribution for each period. Age-standardized rates were calculated using the direct method and the Martinique population census of 1990 as reference. The Martinique population census of 1990 constituted the denominator to calculate HAM/TSP incidence for the first study period 1986–1990. Then populations of 1995, 2000, 2005 and 2010 were used for 1991–1995, 1996–2000, 2001–2005 and 2006–2010 study periods. Standardized incidence rate ratios with 95% CIs and *P* trends were assessed from simple Poisson regression models. *P* for trend was used to either support or reject a linear trend in HAM/TSP standardized incidence rates from the first to the last study period.

Seroprevalence of HTLV-1 within the four periods was expressed as number of seropositive HTLV-1 subjects per one hundred new blood donors. A binomial analysis was used for 95% CI of seroprevalence rates. The first study period (1996–2000) was used as reference to calculate the odds ratio of seroprevalence with 95%CI in the four study periods.

The χ2 test or Fisher-exact test, as appropriate, and Student *t* test or Mann-Whitney test were used to examine differences in nominal and continuous values. All analyses were performed in SAS 9.3 (SAS Institute, Inc, Cary, NC) and MS Excel software.

## Results

**Fig 1:**
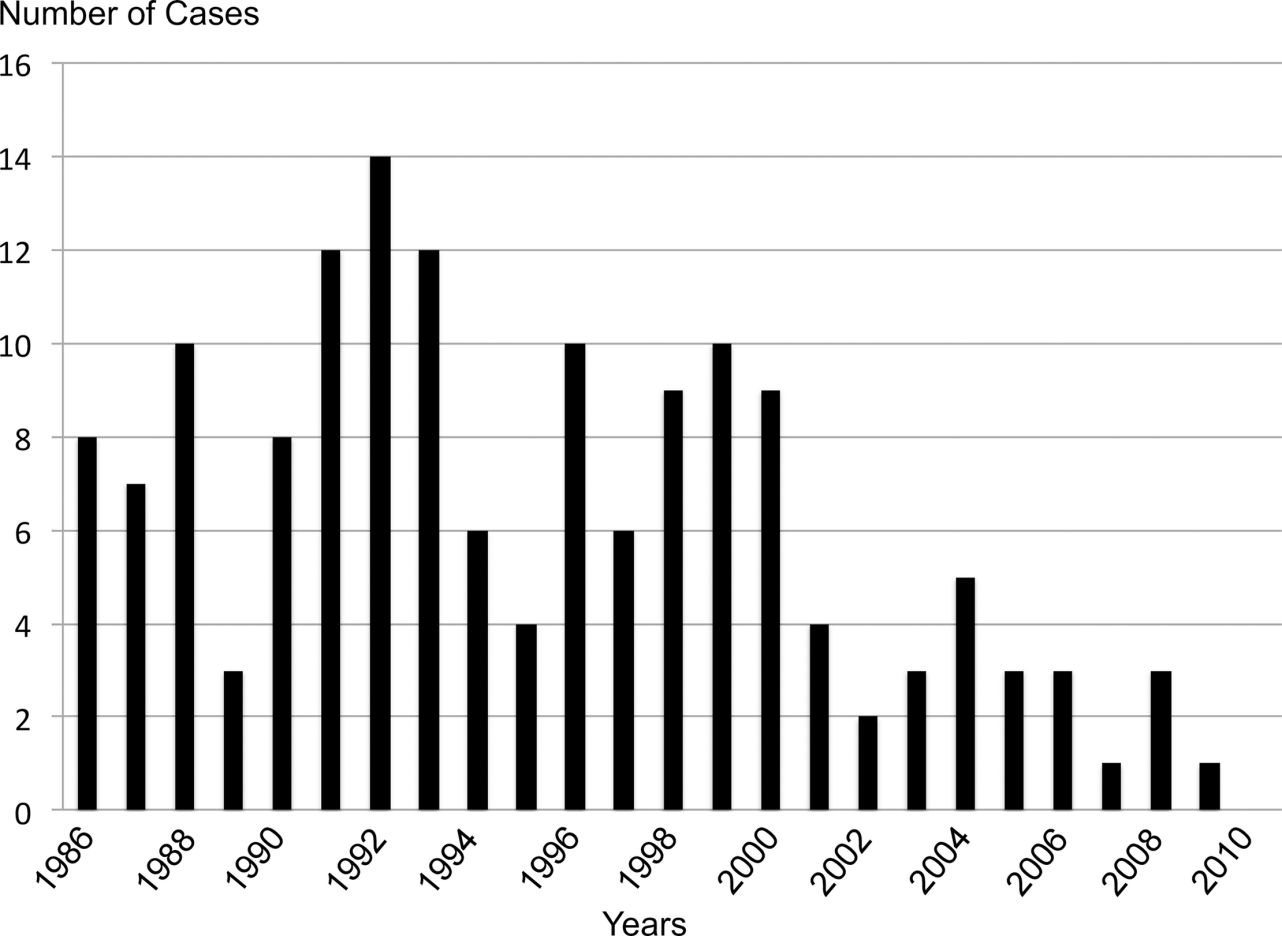
Number of HAM/TSP diagnosis per year over the 25-year study period.

Between January 1986 and June 2015, 326 HAM/TSP patients were included in our registry. We excluded 84 patients who met the diagnosis of probable HAM/TSP according to WHO criteria. Among the 242 definite HAM/TSP patients, 89 were excluded regarding their year of onset before 1986 (83) or after 2010 (6). Eventually, 153 definite HAM/TSP patients experienced a disease onset over the 25-year study period. The mean age at onset was 53 ± 13 years and females predominated (79.7%). Patients experienced gait impairment as first symptoms in 86% and the median onset-diagnosis delay was 3 years. Patient’s characteristics are summarized in Table 1. The Fig 1 shows the number of HAM/TSP diagnosis per year over the 25-year study period.

**Table 1:** HAM/TSP characteristics in the whole cohort.

	Whole cohort N = 153
Age at Onset Mean ± SD (years) Median (years) Range	53±13.1 55 14–77
Sex Ratio F/M (% of female)	122/31 (79.7)
Gait Impairment Onset, n (%)	123 (80.4)
Blood transfusion before 1990, n (%)	32 (20.9)
Onset-to-Diagnosis Delay, Mean ± SD (years) Median (years) Range	4.9±3.2 3 0–14
Proviral load (HTLV-1 copies per 10^6^ PBMCs)	110,794±108,017

PBMCs: peripheral blood mononuclear cells; SD: Standard Deviation.

### Trends in HAM/TSP incidence

The 5-year crude incidence rates (95%CI) per 100,000 in 1986–1990, 1991–1995, 1996–2000, 2001–2005 and 2006–2010 study periods are reported in Table 2. Between 1986 and 2000 rates were stable and ranged from 10.01 (6.78–13.28) to 13.02 (9.34–16.7) whereas rates declined in the two earliest periods 2001–2005 and 2006–2010, 4.27 (2.24–6.28) and 2.03 (0.62–3.43). Age-standardized incidence rates on Martinique population census of 1990 (used for 1986–1990 study period) significantly decreased by 69% and 87% in 2001–2005 and 2006–2010 study periods (Table 2).

**Table 2:** Crude and age-standardized 5-year incidence rates and rate ratio of HAM/TSP in Martinique between 1986 and 2010.

Study Periods	Estimated Population at Risk	Events, n	Crude 5-year Incidence Rate (95%CI)	1986–1990 age-standardized 5-year incidence rates (95%CI)	Standardized Incidence Rate Ratio (95%CI)
**1986–1990**	359,579	36	10.01 (6.78–13.28)	10.01 (6.78–13.28)	1
**1991–1995**	368,735	48	13.02 (9.34–16.70)	11.93 (8.61–15.25)	1.19 (0.87–1.55)
**1996–2000**	381,325	44	11.54 (8.13–14.95)	10.37 (7.28–13.47)	1.04 (0.91–1.13)
**2001–2005**	397,727	17	4.27 (2.24–6.28)	3.13 (1.64–4.62)	0.31 (0.17–0.51)
**2006–2010**	394,171	8	2.03 (0.62–3.43)	1.34 (0.41–2.27)	0.13 (0.09–0.18)

CI indicates Confidence Interval

The 1986-1990-study period was used as reference for the standardized incidence rate ratio calculation.

According to simple Poisson regression model, P for trend of age-standardized 5-year incidence rates over the study periods was calculated as <0.01.

### Trends in HAM/TSP characteristics

Regarding HAM/TSP incidence rate decline from 2001, we compared patient’s characteristics between the two groups whose disease onset was in 1986–2000 and 2001–2010 periods.

Table 3 compares the characteristics of HAM/TSP patients with a disease onset in 1986–2000 and in 2001–2010.

**Table 3:** HAM/TSP characteristics according a disease onset in 1986–2000 and 2001–2010 periods.

	1986–2000 N = 128	2001–2010 N = 25	p
Age at Onset Mean ± SD (years) Median (years) Range	52.1±13.5 55 14–77	57.7±9.6 57 42–72	0.067
Sex Ratio F/M (% of female)	104/24 (81.2)	18/7 (72)	0.43
Gait Impairment Onset, n (%)	104 (81.2)	19 (76)	0.7
Blood transfusion before 1990, n (%)	28 (21.9)	4 (16)	0.59
Onset-to-Diagnosis Delay, Mean ± SD (years) Median (years) Range	5.1±3.1 3 0–14	4.2±3.5 3 1–8	0.45
Proviral load (HTLV-1 copies per 10^6^ PBMCs)	107,954±104,248	135,880±140,000	0.48

Characteristics of 1986–2000 and 2001–2010 study periods were compared using Khi-2 test except for age (Student t-test), Blood transfusion (Fisher’s exact test) and Proviral load (Mann-Whitney test).

Level of HTLV-1 proviral load was missing for 30 patients, 23 HAM/TSP patients with an onset within 1986–2000 study period and 7 within 2001–2010 study period. The other variables were available for all patients.

Sex ratio, initial symptoms, onset-to-diagnosis delay and history of blood transfusion before 1990 were comparable between the two groups. We found a trend to significance for an older age at onset in 2001–2010 incident cases.

Level of HTLV-1 in PBMC was available for 123 out of 153 patients. The mean level of HTLV-1 proviral load did not differed between the two study periods.

### Trends in HTLV-1 infection among first-time blood donors

Table 4 shows the prevalence of HTLV-1 infection in first-time blood donors within the four 5-year study periods. Seropositive male subjects were statistically younger in the first study period compared to the 3 earliest periods. Compared to the first study period (1996–2000), HTLV-1 infection frequencies were significantly lower in the earliest study periods (2001–2005, 2006–2010, 2011–2015) as illustrated by the absence of 95%CI range limits overlap. Between 1996–2000 and 2011–2015 study periods, the HTLV-1 seroprevalence significantly decreased by 63%, 67% and 61% in the whole, female and male population of first-time blood donors respectively.

**Table 4:** Prevalence of HTLV-1 infection among first-time blood donors by genders in the four 5-year study periods.

Study Periods	1996–2000	2001–2005	2006–2010	2011–2015	p
Whole subjects					
First-time Blood Donors, n	11932	12276	12948	9727	
HTLV-1 Seropositive, n	67	46	33	20	
Age (y), Mean±SD	38.7±11.9	43.9±11.2	43.3±13.7	44.1±12.3	0.025
Seroprevalence, %, (95%CI)	0.56 (0.42–0.69)	0.38 (0.27–0.48)	0.26 (0.17–0.34)	0.21 (0.12–0.30)	
Odds Ratio (95%CI)	1	0.67 (0.46–0.98)	0.45 (0.30–0.68)	0.37 (0.22–0.61)	
Female					
First-time blood donors, n	5713	6630	7140	5487	
HTLV-1 Seropositive, n	41	29	24	13	
Age (y), Mean±SD	40.6±11.4	44.1±11.3	43.7±14.6	42.5±15.4	0.35
Seroprevalence, %, (95%CI)	0.72 (0.50–0.94)	0.44 (0.28–0.60)	0.34 (0.20–0.47)	0.24 (0.11–0.37)	
Odds Ratio (95%CI)	1	0.61 (0.38–0.98)	0.47 (0.28–0.78)	0.33 (0.18–0.62)	
Male					
First-time blood donors, n	6219	5646	5808	4240	
HTLV-1 Seropositive, n	26	17	9	7	
Age (y), Mean±SD	35.6±12.2	43.6±11.3	42.4±11.6	46.4±5.7	0.028
Seroprevalence, %, (95%CI)	0.42 (0.26–0.58)	0.30 (0.16–0.44)	0.15 (0.05–0.26)	0.17 (0.04–0.29)	
Odds Ratio (95%CI)	1	0.72 (0.39–1.33)	0.37 (0.17–0.79)	0.39 (0.17–0.90)	

CI indicates Confidence Interval; SD indicates Standard Deviation

Odds ratio were calculated using the 1996–2000 study period as reference

## Discussion

Over 25 years, 1986–2010, our study reports a significant decrease of new definite HAM/TSP cases. Whereas the crude 5-year incidence rates remained stable between 1986 and 2000, ranging from 10.1 to 12.2/100,000, the rates rapidly decline between 2001 and 2010, ranging from 3.2 and 2.9/100,000. The age-standardized 5-year incidence rates exhibit a dramatic decrease by 70% from 2001 and reaching 87% for 2006–2010 periods.

In accordance with previous studies, the age at onset of HAM/TSP patients was around 50 years old [16–18]. We found a trend to a significant older age at onset after 2000 (52.1 versus 57.5, p = 0.06) that may reflect an age cohort effect and that could be indicative of a rapid decrease in HTLV-1 seroprevalence. As usually reported, females are highly predominant in Martinique HAM/TSP patients [19] and the proportion does not differ between 1986–2000 and 2001–2010 onset cases. Blood transfusion constitutes a usual infection route and a study from Japan showed a 16% decrease in the incidence of HAM/TSP 2 years after blood-donor screening implementation [20]. We did not observe any differences in blood transfusion history frequencies between patients whose onset was before or after 1990. As previously reported, [19] initial neurological symptoms were predominantly gait impairment without any difference between the two onset groups. The median delay between onset and diagnosis was stable over the study time and was set at 3 years.

In 1989, HTLV-1 seroprevalence was appraised at 2.2% in the general population of Martinique [21], while it was estimated to 1.93% among pregnant women [22] and to 0.4% among blood donors cohort in the mid-nineties [23]. Although no relevant data are available on HTLV-1 seroprevalence trends in our general population, infection rates in blood donors were analyzed over twenty years from 1996 to 2015. Seroprevalence of HTLV-1 infection among first-time blood donors decreased significantly by 63% between 1996–2000 and 2011–2015 study periods. Seroprevalence decrease may partly account for HAM/TSP incidence decline and may result from several factors. These include the rapid westernization of life style that spread through the whole population from the eighties, the systematic screening for HTLV-1 antibodies in volunteer blood donor and pregnant women [16,17] that has been implemented in the early nineties in Martinique and the iterative information campaigns for HIV and HTLV-1 prevention that particularly encouraged the use of condoms in the population. Prevention strategy leading to refrain HTLV-1 infected mother to breast fed their children has demonstrated efficiency in seroprevalence decrease [9]. However, such a dramatic HAM/TSP incidence decline from 2001 is intriguing. Indeed, individuals aged around 50 years old between 2001 and 2010 became sexually active before implementation of HTLV-1 transmission prevention strategy. Additionally, they were all exposed to HTLV-1 contamination through breastfeeding and were at exposure risk through untested blood transfusion at least the first 20 years of life. HTLV-1 transmission prevention strategy could not be sufficient to account for such an early incidence decrease. This raises the question of a co-factor that could play a role in refraining the development of HAM/TSP disease among HTLV-1 infected population. Rapid and extensive westernization improved the socioeconomic level of the Martinique population and was followed by sanitary changes to the environment. Sanitation and hygienic behavior improvements resulted in a rapid reduction in parasite infection. Between 1978 and 1994, the intestinal parasitism in children decreased from 70% to 8% [24]. Few studies, with divergent conclusions, focused on HAM/TSP development and parasite infection. Whereas a study assume that *Schistosoma* proteins may reduce inflammatory process associated with HTLV-1 infection through interferon-gamma level decrease [25], another one report that treatment of helminthic infection does not affect the risk of HAM/TSP development [26]. Gillet et al. [27] suggest that co-infection with *Strongyloides* is associated with formation of new HTLV-1-infected T-cell clones and with oligoclonal proliferation of certain HTLV-1 clones, increasing thereby the risk of HTLV-1 disease expression. On the other hand, whereas HTLV-1 and *Strongyloides* infect 43% and 35% of tested indigenous in Central Australia [28], the HAM/TSP prevalence appears very low in these communities with extremely poor access to sanitation and hygiene facilities [29]. Studies are still needed to investigate the role of a potential co-factor associated with sanitary change and potentially leading to decline in number of HAM/TSP diagnosis. The present study does not allow reliable conclusions about the synergistic effects of HTLV-1 transmission prevention programs and improvement of sanitary conditions in HAM/TSP incidence rates decline from 2001 in Martinique.

Whereas meta-analyses show no clear evidence that structural interventions at community level, to increase condom use, prevent the transmission of HIV and other sexually transmitted infections [30], no reliable study has focused on HTLV-1 infection. We assume that HTLV-1 sexual transmission is probably the next challenge in Martinique to continue HTLV-1 seroprevalence decrease. Prevention strategies focusing on sexual transmission have to be intensified and should lead to increase acceptability of condom in the general population using mass media campaigns, public opinion, meeting’s school information and social marketing advertisement; improve condom accessibility particularly for low-income population; improve female access to condom; extend the availability of condoms such as condom machine installation in public and private spaces.

Despite the large study period of 25 years, the number of new HAM/TSP cases remains limited with low statistical power particularly in comparing the characteristics of the two groups of patients with a disease onset before and after 2001. Between 1986 and 2015, technological improvement of imaging and laboratory investigations may have influenced HAM/TSP diagnosis accuracy. However, HAM/TSP diagnosis criteria did not change during the study time and imaging methods that are crucial to rule out alternative myelopathy and especially spinal cord compression, were available through the whole study time. A reduction of HAM/TSP patients referred to our network by the primary health setting over the study period might be raised. However, only slight changes occurred to the global health care network for HTLV-1 infected patients. Moreover, we assume that improvement of medical management of HAM/TSP patients in Neurology and Rehabilitation centers may have prompted general practitioners and primary care centers to refer more systematically patients to our network. We showed that HTLV-1 seroprevalence have decreased over 20 years among first-time blood donors, but the absence of demographic characteristics of this population limits our findings interpretation.

In summary, Martinique faces a rapid and sustained decline of HAM/TSP incidence from 2001 estimated by 70% in comparison to 1986–2000 periods. Implementation of HTLV-1 transmission prevention strategy may result in seroprevalence reduction and in HAM/TSP incidence decrease observed 15 years later. The monitoring of new HAM/TSP cases has to be continued and HTLV-1 seroprevalence in blood donors and pregnant women must be sequentially assessed.

## Supporting information

S1 Checklist STROBE checklist. (DOC)

S1 Data Excel data files. (XLSX)
