# Supplementary material for: Temporal trends in Human T-Lymphotropic virus 1 (HTLV-1) associated myelopathy/tropical spastic paraparesis (HAM/TSP) incidence in Martinique over 25 years (1986-2010)
Source: PLoS Negl Trop Dis. 2018 Mar 19;12(3):e0006304. doi: 10.1371/journal.pntd.0006304 (PMC5875895; doi:10.1371/journal.pntd.0006304)
Supplement: S1 Checklist — (DOC) [file pntd.0006304.s001.doc]

STROBE Statement—Checklist of items that should be included in reports of ***cohort studies***

|  | Item No | Recommendation |
| --- | --- | --- |
| **Title and abstract** | 1 | (*a*) Indicate the study’s design with a commonly used term in the title or the abstract : Abstract Section Methods |
|  |  | (*b*) Provide in the abstract an informative and balanced summary of what was done and what was found: Abstract |
| Introduction | | |
| Background/rationale | 2 | Explain the scientific background and rationale for the investigation being reported: Introduction |
| Objectives | 3 | State specific objectives, including any prespecified hypotheses: Introduction, last paragraph |
| Methods | | |
| Study design | 4 | Present key elements of study design early in the paper: Section Methods, Sub-section “study design and population” |
| Setting | 5 | Describe the setting, locations, and relevant dates, including periods of recruitment, exposure, follow-up, and data collection: Section methods, sub sections “study design…” and “Case ascertainment…” |
| Participants | 6 | (*a*) Give the eligibility criteria, and the sources and methods of selection of participants. Section Methods, sub-section “Case ascertainment…” 1^st^ and last paragraph. Describe methods of follow-up : Not applicable |
|  |  | (*b*) For matched studies, give matching criteria and number of exposed and unexposed: Not applicable |
| Variables | 7 | Clearly define all outcomes, exposures, predictors, potential confounders, and effect modifiers. Section Methods, Sub-section “study design and population”, 2^nd^ paragraph. Give diagnostic criteria, if applicable: Section Methods, sub-section “Case ascertainment…”, last paragraph |
| Data sources/ measurement | 8* | For each variable of interest, give sources of data and details of methods of assessment (measurement). Section Methods, sub-section “Case ascertainment…” and sub-section “HTLV-1 seroprevalence assessment…” Describe comparability of assessment methods if there is more than one group: Section Methods, Sub-section “Statistical Analysis”, 2^nd^ Paragraph. |
| Bias | 9 | Describe any efforts to address potential sources of bias Section Methods, sub-section “Case ascertainment…” 1^st^ paragraph |
| Study size | 10 | Explain how the study size was arrived at Section Methods, sub-section “Case ascertainment…” 1^st^ paragraph |
| Quantitative variables | 11 | Explain how quantitative variables were handled in the analyses. If applicable, describe which groupings were chosen and why : Section Methods, Sub-Section “Statistical analysis”, first Paragraph |
| Statistical methods | 12 | (*a*) Describe all statistical methods, including those used to control for confounding: Section Methods, Sub-section “Statistical Analysis” |
|  |  | (*b*) Describe any methods used to examine subgroups and interactions: Section Methods, Sub-section “Statistical Analysis” |
|  |  | (*c*) Explain how missing data were addressed: Proviral load was the only studied variable with missing data. Not reported in Methods Section but in Legend of Table 1 in Results Section. |
|  |  | (*d*) If applicable, explain how loss to follow-up was addressed: Not Apllicable |
|  |  | (*e*) Describe any sensitivity analyses: Population age Adjustment for incidence rate over the study constituted the major sensitivity analysis to confirm rate decrease. Section Maethods, Sub-Section “Statistical Analysis” |
| Results | | |
| Participants | 13* | (a) Report numbers of individuals at each stage of study—eg numbers potentially eligible, examined for eligibility, confirmed eligible, included in the study, completing follow-up, and analysed : Section Results, 1^st^ Paragraph. |
|  |  | (b) Give reasons for non-participation at each stage: Section Results, 1^st^ Paragraph |
|  |  | (c) Consider use of a flow diagram: Not used in the present study |
| Descriptive data | 14* | (a) Give characteristics of study participants (eg demographic, clinical, social) and information on exposures and potential confounders: Section Results table 1. |
|  |  | (b) Indicate number of participants with missing data for each variable of interest : Legend of Table 1 |
|  |  | (c) Summarise follow-up time (eg, average and total amount): Not Applicable |
| Outcome data | 15* | Report numbers of outcome events or summary measures over time: Not Applicable |
| Main results | 16 | (*a*) Give unadjusted estimates and, if applicable, confounder-adjusted estimates and their precision (eg, 95% confidence interval). Make clear which confounders were adjusted for and why they were included: Section Results, sub sections “Trends in HAM/TSP incidence” and “Trends in HTLV-1 infection….” And Tables 2 and 3. |
|  |  | (*b*) Report category boundaries when continuous variables were categorized: Not Applicable |
|  |  | (*c*) If relevant, consider translating estimates of relative risk into absolute risk for a meaningful time period: Not Applicable |
| Other analyses | 17 | Report other analyses done—eg analyses of subgroups and interactions, and sensitivity analyses: Not Applicable |
| Discussion | | |
| Key results | 18 | Summarise key results with reference to study objectives: Section Discussion, First Paragraph |
| Limitations | 19 | Discuss limitations of the study, taking into account sources of potential bias or imprecision. Discuss both direction and magnitude of any potential bias: Section Discussion 4^th^ Paragraph |
| Interpretation | 20 | Give a cautious overall interpretation of results considering objectives, limitations, multiplicity of analyses, results from similar studies, and other relevant evidence: Section Discussion 2^nd^ and 3^rd^ Paragraph |
| Generalisability | 21 | Discuss the generalisability (external validity) of the study results : Summary paragraph of Section Discussion. |
| Other information | | |
| Funding | 22 | Give the source of funding and the role of the funders for the present study and, if applicable, for the original study on which the present article is based: Observational study without specific funding |

*Give information separately for exposed and unexposed groups.

**Note:** An Explanation and Elaboration article discusses each checklist item and gives methodological background and published examples of transparent reporting. The STROBE checklist is best used in conjunction with this article (freely available on the Web sites of PLoS Medicine at http://www.plosmedicine.org/, Annals of Internal Medicine at http://www.annals.org/, and Epidemiology at http://www.epidem.com/). Information on the STROBE Initiative is available at http://www.strobe-statement.org.
